# Higher Protein Kinase C ζ in Fatty Rat Liver and Its Effect on Insulin Actions in Primary Hepatocytes

**DOI:** 10.1371/journal.pone.0121890

**Published:** 2015-03-30

**Authors:** Wei Chen, Matthew Ray Goff, Heqian Kuang, Guoxun Chen

**Affiliations:** Department of Nutrition, University of Tennessee at Knoxville, Knoxville, Tennessee, United States of America; East Tennessee State University, UNITED STATES

## Abstract

We previously showed the impairment of insulin-regulated gene expression in the primary hepatocytes from Zucker fatty (ZF) rats, and its association with alterations of hepatic glucose and lipid metabolism. However, the molecular mechanism is unknown. A preliminary experiment shows that the expression level of protein kinase C ζ (PKCζ), a member of atypical PKC family, is higher in the liver and hepatocytes of ZF rats than that of Zucker lean (ZL) rats. Herein, we intend to investigate the roles of atypical protein kinase C in the regulation of hepatic gene expression. The insulin-regulated hepatic gene expression was evaluated in ZL primary hepatocytes treated with atypical PKC recombinant adenoviruses. Recombinant adenovirus-mediated overexpression of PKCζ, or the other atypical PKC member PKCι/λ, alters the basal and impairs the insulin-regulated expressions of glucokinase, sterol regulatory element-binding protein 1c, the cytosolic form of phosphoenolpyruvate carboxykinase, the catalytic subunit of glucose 6-phosphatase, and insulin like growth factor-binding protein 1 in ZL primary hepatocytes. PKCζ or PKCι/λ overexpression also reduces the protein level of insulin receptor substrate 1, and the insulin-induced phosphorylation of AKT at Ser473 and Thr308. Additionally, PKCι/λ overexpression impairs the insulin-induced *Prckz* expression, indicating the crosstalk between PKCζ and PKCι/λ. We conclude that the PKCζ expression is elevated in hepatocytes of insulin resistant ZF rats. Overexpressions of aPKCs in primary hepatocytes impair insulin signal transduction, and in turn, the down-stream insulin-regulated gene expression. These data suggest that elevation of aPKC expression may contribute to the hepatic insulin resistance at gene expression level.

## Introduction

The normal physiological responses to insulin stimulation in the liver include the increase of glycolysis and lipogenesis, and the reduction of gluconeogenesis [1,2]. In hepatic parenchymal cells, insulin initiates a signaling cascade upon binding to its receptor on the cell membrane, which is followed by the activation of insulin receptor substrates (IRSs) [3]. Tyrosine phosphorylation of IRS1/2 leads to the association and activation of phosphatidylinositol 3-kinase (PI3K). PI3K catalyzes the conversion of phosphatidylinositol-4,5-bisphosphate into phosphatidylinositol-3,4,5-triphosphate. The latter is a lipid messenger that anchors the downstream effector proteins, e.g. phosphoinositide dependent protein kinase-1, AKT (also known as protein kinase B) and atypical protein kinase C (aPKC), to the cell membrane [3]. For its maximal activation, AKT is phosphorylated by 3-phosphoinositide-dependent kinase 1 (PDK1) at Thr308, and by mammalian target of rapamycin complex 2 (mTORC2) at Ser473 [4]. Replacing either one of them with alanine residue will significantly impair the insulin-induced AKT activity [5]. The activations of AKT and aPKC differentially regulate the transcription of hepatic genes for glucose and lipid metabolism [6–8]. For example, the activation of AKT induces the expression of glucokinase (GCK, gene *Gck*) to promote glycolysis, and suppresses the expression of cytosolic form of phosphoenolpyruvate carboxykinase (PEPCK, gene *Pck1*) to reduce gluconeogenesis [9]. On the other hand, the insulin-induced expression of sterol-responsive element binding protein 1c (SREBP-1c, gene *Srebp-1c*) has been attributed to the activations of AKT [10,11] and aPKC [12,13].

Members of PKC family include classical PKC, novel PKC, and aPKC. Unlike the other two types of PKC, aPKC does not require diacylglycerol or Ca^2+^ for activation. PKCζ and PKCι/λ (genes *Prkcz* and *Prkci*, respectively) are two current members of the aPKC subfamily. PKCζ shares 70% homology in amino acid sequence with PKCι/λ (PKCι in human; PKCλ in mouse) [6]. Both PKCζ and PKCι/λ mediate a variety of physiological events, such as cell polarity establishment, cell motility, immune response and metabolism [4].

Activation of PKCζ or PKCι/λ has been associated with increased lipogenesis and insulin resistance in multiple tissues and organs [6,14]. The aPKC activation seems to regulate the *Srebp-1c* transcription and lipogenesis in the mouse liver. For example, in liver-specific PKCλ knockout mice, the basal transcript level of *Srebp-1c*, but not *Gck* or *Pck1*, declined by 50% [12]. In mice with the liver-specific deletion of the regulatory subunit of PI3K, PKCζ/λ activity decreased in association with the reduction of *Srebp-1c* expression [8]. On the other hand, the insulin-induced activation of aPKC and subsequent *Srebp-1c* expression were retained in the liver of diabetic mice [15,16]. PKCζ/λ is also activated in the liver of mice fed a high fat diet, which is accompanied by the elevated expression of hepatic *Srebp-1c* [13]. This effect could be negated by the overexpression of kinase-inactive form of PKCζ [13]. All these observations suggest the role of aPKCs in the regulation of hepatic lipogenesis.

In hepatocytes isolated from individuals with type 2 diabetes, the basal and insulin-induced activation of PKCι were elevated [17,18]. Treatment of these hepatocytes with PKCι inhibitors lowered the expression levels of lipogenic, proinflammatory and gluconeogenic enzymes [17,18]. Moreover, metformin treatment was shown to induce aPKC activities, and increase lipogenic and gluconeogenic enzyme levels in hepatocytes from non-obese human subjects [19]. These data collectively suggest that aPKC isoforms are important players in the pathogenesis of obesity and type 2 diabetes. However, whether aPKC plays a role in the insulin-regulated gene expression in hepatocytes is still unclear.

Zucker fatty (ZF) rat is a genetic model of obesity, hypertriglyceridemia and hepatic insulin resistance [20]. We previously showed that primary hepatocytes from ZF rats fed chow ad libitum exhibited impairment of the insulin-regulated gene expression [21]. Interestingly, the insulin-induced phosphorylation of AKT at Ser473 and Thr308 in primary hepatocytes of ZF rats was not significantly different from that of Zucker lean (ZL) rats [21]. Therefore, we systemically compared the activation states of other components of insulin signal transduction pathway between ZL and ZF hepatocytes. Here, we report the differential expression levels of PKCζ in the liver of ZF and ZL rats, and the effects of PKCζ and PKCι/λ overexpression on the insulin-regulated gene expression in primary rat hepatocytes. We demonstrated that overexpression of PKCζ or PKCι/λ in ZL primary hepatocytes impaired the insulin-regulated gene expression.

## Materials and Methods

### Reagents

The reagents for primary hepatocyte isolation and culture including Medium 199, Dulbecco’s Modification of Eagle Medium (DMEM), liver perfusion buffer and liver digest buffer were obtained from Invitrogen (Carlsbad, CA). For RNA extraction, RNA STAT-60 was purchased from TEL-TEST (Friendswood, TX). The reagents for cDNA synthesis and real-time PCR were obtained from Applied Biosystems (Foster city, CA). All real-time PCR primer sets used in this study were synthesized by Sigma-Aldrich (St. Louis, MO). Antibodies against β-actin (#4967), PKCζ (#9368), PKCι (#2998), phospho- PKCζ/λ (p-PKCζ/λ) Thr410/403 (#9378), AKT (#9272), phospho-AKT (p-AKT) Ser473 (#9271), p-AKT Thr308 (#9275), p-AKT Thr450 (#9267), insulin receptor substrate 1 (IRS1, #2382), fatty acid synthase (FAS, #3189), acetyl CoA carboxylase (ACC, #3662), phospho-ACC (p-ACC) Ser79 (#3661) used in this study were purchased from Cell Signaling Technology (Danvers, MA). All other reagents and materials were purchased from Fisher Scientific (Pittsburgh, PA) unless described otherwise.

### Animals and diets

Zucker rats were bred and housed under constant temperature and humidity in the animal facility on a 12-hour light-dark cycle. Male ZL (fa/+ or +/+) or ZF (fa/fa) rats at weaning (3 weeks old) were kept on Teklad rodent chow *ad libitum* (#8640, Harlan Laboratories, Indianapolis, IN) for 8 weeks before liver tissue collection and primary hepatocyte isolation. All procedures were approved by the Institutional Animal Care and Use Committee at the University of Tennessee at Knoxville (Protocols #1256, 1642 and 1863).

### Liver tissue collection, total protein preparation and total RNA extraction

The rat was euthanized by primary carbon dioxide asphyxiation, and then secondary cervical dislocation according to regulations. The procedure for liver tissue collection was reported elsewhere [21,22]. In brief, a 10ml syringe with a 21G × 1½" hypodermic needle was used to drain blood from the liver via the inferior vena cava. The liver was excised, sliced, then snap-frozen in liquid nitrogen, and store at -80°C before further analysis. A small portion of the liver tissue was homogenized in 10 volumes of cold whole-cell lysis buffer (1% Triton X-100, 10% glycerol, 1% IGEPAL CA-630, 50mM Hepes, protease inhibitors, pH 8.0), and then centrifuged to remove insoluble matters [21,22]. The protein concentration of the supernatant was determined with PIERCE BCA protein assay kit. Another small portion of the liver tissue was homogenized in 10 volumes of cold STAT-60. Total RNA was extracted according to the manufacturer instructions.

### Cultures of primary hepatocytes and 293 HEK cells

The primary hepatocytes were isolated according to published methods [23]. Isolated hepatocytes were seeded on 60-mm collagen type I coated dishes at 2×10^6^ cells per dish and incubated in DMEM (4.5g/L glucose, 8% FBS, 1% penicillin/streptomycin) for 3 hours to allow cell attachment. The attached primary hepatocytes were washed once with PBS and pretreated in medium A (Medium 199 with 100nM dexamethasone, 100nM 3,3’,5-triiodo-L-thyronine, 1nM insulin, and 1% penicillin/streptomycin) for 16–18 hours. HEK 293 cells were seeded and kept in DMEM containing 4.5g/L glucose, 4% FBS, 1% penicillin/streptomycin [23].

### Cloning of the rat *Prkci* cDNA and subcloning of the rat *Prkcz* cDNA

Based upon the rat *Prkci* mRNA sequence (GenBank: EU517502.1), sense 5’-ATC CCC TCA GCC TCC AGC GG-3’ and antisense 5’-ACT GTG ACC GGG CTA ACG GT-3’ primers were designed using Primer-BLAST tools from the National Center Biotechnology Information. For the complete coding sequence of rat *Prkci* cDNA, PCR was carried out using cDNA derived from total RNA of ZL primary hepatocyte as the template. For subcloning of rat *Prkcz*, pEYFP-N1 vector containing its complete coding sequence (generous gift from Dr. Ralf Kubitz) was used as the template for the PCR amplification of *Prkcz* with sense primer 5’-ACC TCG AGA TGC CCA GCA GGA CCG AC-3’ and antisense primer 5’- GTG AAT TCA CAC GGA CTC CTC AGC AGA C-3’. The amplicons containing the complete coding sequences of *Prkci* and *Prkcz* cDNA sequences were ligated into pCR2.1 vector through TA Cloning Kit (Invitrogen) according to the manufacturer’s protocol.

### Generation of Ad-Prkcz and Ad-Prkci recombinant adenoviruses

The inserts containing the complete coding sequences of *Prkcz* and *Prkci* were subcloned into pACCMV5 vector to make pACCMV5-Prkcz and pACCMV5-Prkci, respectively. To generate Ad-Prkcz and Ad-Prkci recombinant adenoviruses, pACCMV5 plasmid containing the complete coding sequence of atypical *Prkc* sequences was co-transfected with JM17 into HEK293 cells using FuGENE^®^6 Transfection Reagent (Roche) according to manufacturer instructions. After the formation of the viral plaques, the crude lysate was collected and stored at -80°C. The amplification and purification of adenovirus were carried out according to published protocols [23]. The optical density (OD) at 260nm of the adenoviral suspension was determined to estimate the plaque forming units (pfu) of the purified recombinant adenoviruses. We used that 1 OD equals to 1 × 10^12^ pfu/ml. The purified recombinant adenoviruses were stored at -80°C until being used.

### Infection of recombinant adenoviruses and treatments of primary hepatocytes

In experiments using recombinant adenoviruses, purified Ad-Prkcz and Ad-Prkci were added in the medium A (5,000 pfu/cell) to allow the overexpression of PKCζ and PKCι/λ in the primary rat hepatocytes during the pretreatment period, respectively. The pretreated hepatocytes were then washed once with PBS, and treated with medium A containing indicated concentrations of insulin (0nM to 100nM) for 6 hours before total RNA extraction, or for 15 minutes before whole cell lysate preparation.

### RNA extraction and real-time PCR analysis

The methods for total RNA extraction and cDNA synthesis were described elsewhere [21]. The gene expression level was determined by real-time PCR with respective primer sets, and normalized to the mRNA level of ribosomal gene 36B4. The nucleotide sequences of primer sets were as follows: *Gck*, 5′-CGA GAT GCT ATC AAG AGG AGA G-3′ (forward), 5′-TCA CA T TGG CGG TCT TCA TAG-3′ (reverse); *Pck1*, 5′-AGT CAC CAT CAC TTC CTG GAA GA-3′ (forward), 5′-GGT GCA GAA TCG CGA GTT G-3′ (reverse); *Srebp-1c*, 5′-GGA GCC ATG GAT TGC ACA TT-3′ (forward), 5′-AGG CCA GGG AAG TCA CTG TCT-3′ (reverse); *Pklr*, 5′-CGT TTG TGC CAC ACA GAT GCT-3′ (forward), 5′-CA T TGG CCA CAT CGC TTG TCT-3′ (reverse); *G6pc*, 5′-GAC CTC AGG AAC GCC TTC TAT G-3′ (forward), 5′-ATT GAT GCC CAC AGT CTC TTG A-3′ (reverse); *Igfbp-1*, 5′-GAT CAC TGA CCT CAA GAA ATG GAA-3′ (forward), 5′-GCG GCA CGT TAA TCT CTC TAA CA-3′ (reverse). The data were presented as either minus Δ cycle threshold (Ct) or the induction fold (ΔΔCt) for which the control treatment group was arbitrarily set as 1.

### Immunoblot analysis of proteins

Protein samples (40μg) of the whole cell lysates of primary hepatocytes or liver tissues were resolved in 8% SDS polyacrylamide gel, and then transferred to BIO-RAD Immuno-Blot PVDF membrane (Hercules, CA) as described [23]. Membranes were blocked by 8% non-fat milk, and then probed with specific antibodies (1:1 000 dilution). After gentle wash, membranes were incubated with goat anti-rabbit IgG conjugated with horseradish peroxidase (1:5,000 dilution). After washing, antigen-bound antibody was detected using ECL Western Blotting Substrate (Thermo Scientific), and subsequently exposure to X-ray films. The films were scanned, and the images were stored for the densitometry analysis using ImageJ software (NIH). Densitometry data for each protein were normalized to β-actin levels in each sample.

### Statistical Analysis

Statistical analyses were performed using SPSS 19.0 software. Student t-test was used to compare the means between two groups. One-way ANOVA with LSD post-hoc test was used to compare the means among three or more groups. Data were presented as means ± S.E.M. The number of experiments indicates hepatocyte isolations from different animals. A p value less than 0.05 is considered statistically significant.

## Results

### PKCζ expression is elevated in the liver of ZF rats

**Fig 1:**
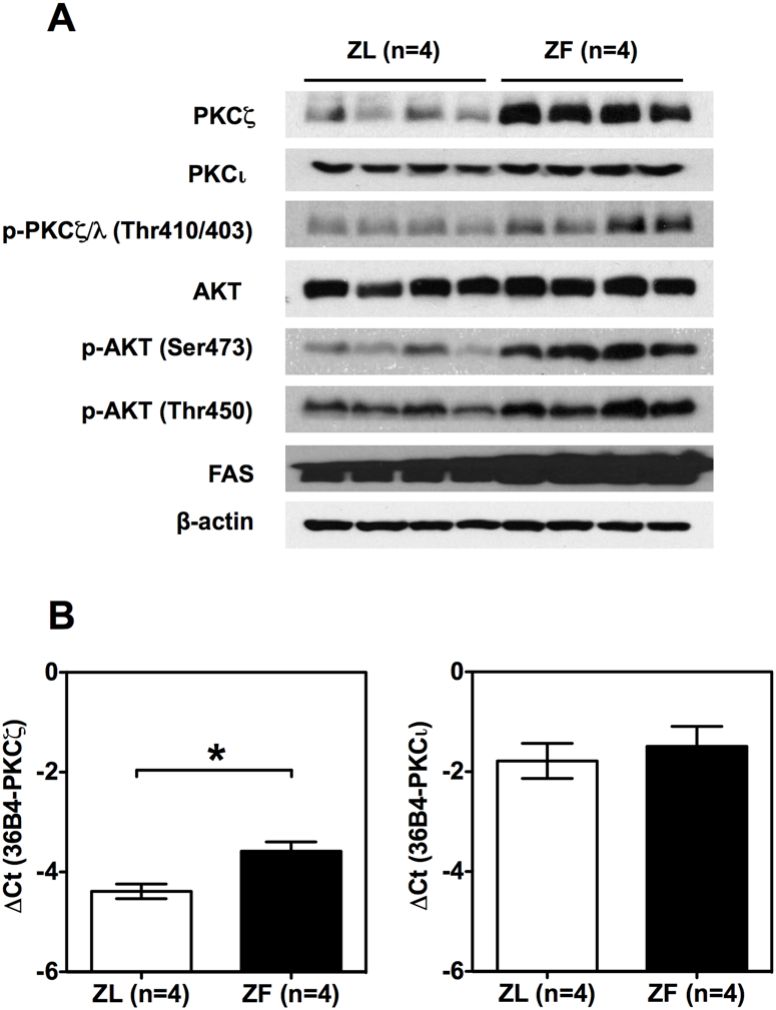
ZF rats fed chow ad libitum had elevation of expression levels of PKCζ mRNA and protein in the liver. (A) Immunoblot analysis of the expression levels of the perspective proteins in the liver of ZL (n = 4) and ZF rats (n = 4). (B) The mRNA levels of *Prkcz* and *Prcki* in the liver of ZL and ZF rats. ZL and ZF rats at weaning (3 weeks old) were fed chow diet for 8 weeks. Liver samples of rats fed in ad libitum were collected and subjected to analysis of protein and mRNA levels as described in the Materials and Methods. The data were presented mean ± SEM of -ΔCt with animal numbers in parenthesis (* indicates p<0.05 for comparing ZL and ZF using Student’s t-test).

Fig. 1A shows that, despite the well-reported hepatic insulin resistance, the levels of phospho-AKT at Ser473 and Thr450 in the liver of ZF rats were higher than that of ZL rats (please see densitometry analysis in S1 Fig.). The phospho-AKT at Thr308 in the liver tissues could not be detected in the current experimental condition, probably due to non-maximal activation of AKT in ad libitum state, relative less AKT protein amount from hepatocytes in the liver tissue lysate or rapid de-phosphorylation of AKT at Thr308 during the process of euthanasia of the animals. This observation shows that the AKT phosphorylation at Ser473 and Thr450 in ZF rat liver tissue is unimpaired, suggesting the consequence of hyperinsulinemia. In addition, ZF rat liver had higher protein level of FAS, indicating elevation of hepatic lipogenesis.

On the other hand, the protein level of PKCζ, but not PKCι/λ, in the liver of ZF rats was higher than that of ZL rats (Fig. 1A). The level of phospho-PKCζ/λ at Thr410/403, which indicated the phosphorylation of both PKCζ and PKC ι/λ, was higher in the liver samples of ZF rats than that of ZL rats (Fig. 1A). The mRNA level of *Prkcz*, but not that of *Prkci*, was also elevated in the ZF liver (Fig. 1B). These data collectively demonstrated that the aPKC expression levels changes with the development of hepatic insulin resistance in the ZF liver.

### Short-term insulin treatment did not alter protein levels of total and phospho-aPKCs in primary rat hepatocytes

**Fig 2:**
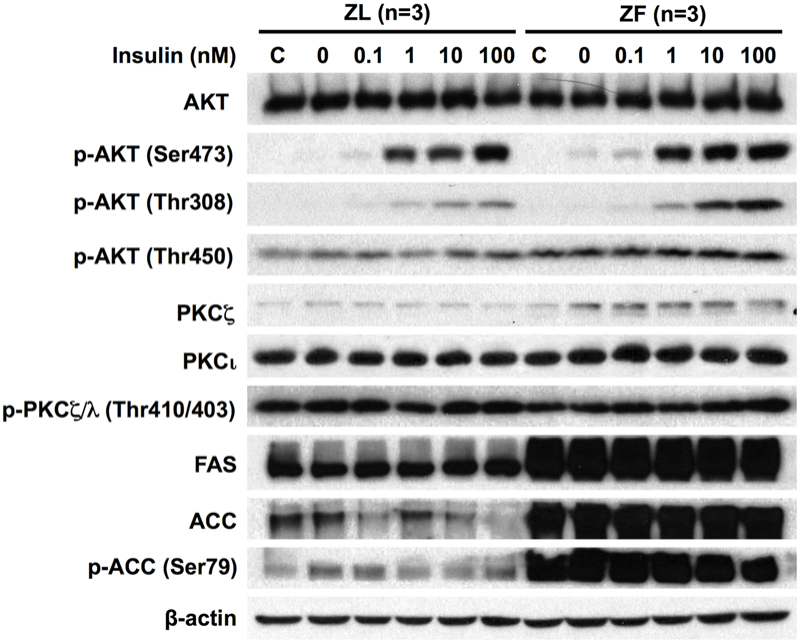
Acute insulin treatment did not alter protein levels of total and phospho-aPKCs in primary hepatocytes from ZL and ZF rats. The primary hepatocytes from ZL and ZF rats (chow diet, 8 weeks) were pre-treated as described in the Materials and Methods for overnight, and then incubated in fresh medium A with increasing concentrations of insulin (0nM to 100nM) for 15 min. The hepatocyte group remained in pre-treatment medium was indicated by “C”. Immunoblot analysis with antibodies against the indicated proteins was performed. The figure shows the representative result of three parallel experiments obtained from three sets of primary hepatocytes independently isolated from ZL and ZF rats fed chow diet ad libitum.

Fig. 2 shows that protein levels of FAS, ACC and phospho-ACC at Ser79 in hepatocytes of ZF rats were higher than that of ZL rats. This result demonstrates that the hepatic lipogenic capability remains elevated in ZF hepatocytes cultured for 20 hours. Insulin treatment dose-dependently induced the levels of phospho-AKT at Ser473 and Thr308 in ZL and ZF primary hepatocytes similarly (Fig. 2). The level of phospho-AKT at Thr450, which was insulin-independent, was slightly higher in ZF hepatocytes than in ZL hepatocytes (Fig. 2). These data demonstrated that the insulin-induced AKT phosphorylation is comparable in both ZL and ZF rat hepatocytes. The impaired insulin-regulated gene expression in ZF hepatocytes may be caused by changes in other components of insulin signaling pathway. On the other hand, the 15-minute insulin treatment did not significantly induce or suppress the protein levels of PKCζ and PKCι/λ and phospho-PKCζ/λ at Thr410/403 in ZL and ZF primary hepatocytes (Fig. 2). There was a slight induction of the total PKCζ when fresh M199 was added. ZF primary hepatocytes had higher level of PKCζ than ZL primary hepatocytes (Fig. 2), which was in line with the finding from the liver tissues (Fig. 1).

### Overexpressions of PKCζ and PKCι/λ in ZL primary hepatocytes

**Fig 3:**
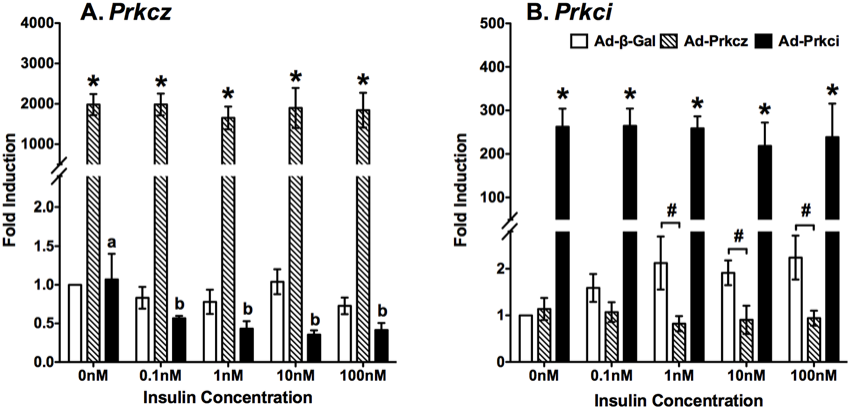
Overexpression of PKCζ and PKCι/λ in primary hepatocytes of ZL rats. Primary hepatocytes of ZL rats fed chow for 8 weeks were isolated and seeded onto dishes as described in the Materials and Methods. Purified Ad-β-gal, Ad-Prkcz and Ad-Prkci were added in medium A during the overnight pretreatment period to allow the overexpression of β-GAL, PKCζ and PKCι/λ, respectively. The primary hepatocytes were then incubated in fresh medium A with increasing concentrations of insulin (0 nM to 100 nM) for 6 hours before total RNA extraction. Total RNA was extracted, and then subjected to real-time PCR analysis. The data were expressed as fold induction. The gene transcript levels from Ad- β-gal infected primary hepatocytes without insulin treatment were arbitrarily set to 1. (A) The mRNA levels of *Prkcz* in the ZL primary hepatocytes overexpressing β-GAL, PKCζ and PKCι/λ in response to treatments of increasing concentrations of insulin (mean ± SEM; n = 4; all p<0.05; a>b for comparing insulin treatment groups in hepatocytes overexpressing PKCζ using one-way ANOVA; * for comparing fold inductions among three groups at indicated treatments using one-way ANOVA). (B) The mRNA levels of *Prkci* in the ZL primary hepatocytes (n = 4) overexpressing β-GAL, PKCζ and PKCι/λ in response to treatments of increasing concentrations of insulin (mean ± SEM; n = 4; all p<0.05; * and # for comparing fold inductions among three groups at indicated treatments using one-way ANOVA).

To investigate whether elevated aPKC protein levels contribute to the hepatic insulin resistance at gene expression level, we constructed recombinant adenoviruses Ad-Prkcz and Ad-Prkci for their overexpressions in ZL primary rat hepatocytes. The expression level of *Pckcz* or *Prkci* mRNA was normalized to that of ribosomal gene 36B4, which was stable across all treatment groups (S2 Fig.). In hepatocytes transfected with Ad-β-gal, the relative abundance of *Prkcz* mRNA (-ΔCt of -7.90) was much lower than that of *Prkci* mRNA (-ΔCt of -4.20). The overexpression fold of *Pckcz* or *Prkci* was calculated using their levels in the Ad-β-gal group as 1. Ad-Prkcz and Ad-Prkci respectively caused overexpressions of *Prkcz* mRNA (-ΔCt of 3.13, ~2 000 fold, Fig. 3A) and *Prkci* mRNA (-ΔCt of 3.78, ~250 fold, Fig. 3B, *).

Interestingly, 0.1-100nM insulin treatments significantly suppressed the expression levels of *Prkcz* mRNA by 40–60% in hepatocytes transfected with Ad-Prkci (Fig. 3A, black columns). On the other hand, in hepatocytes transfected with Ad-Prkcz, the expression levels of *Prkci* mRNA in 1-100nM insulin treatment groups were significantly lower than that in hepatocytes with β-GAL overexpression at the corresponding treatments (Fig. 3B, striped columns, #).

**Fig 4:**
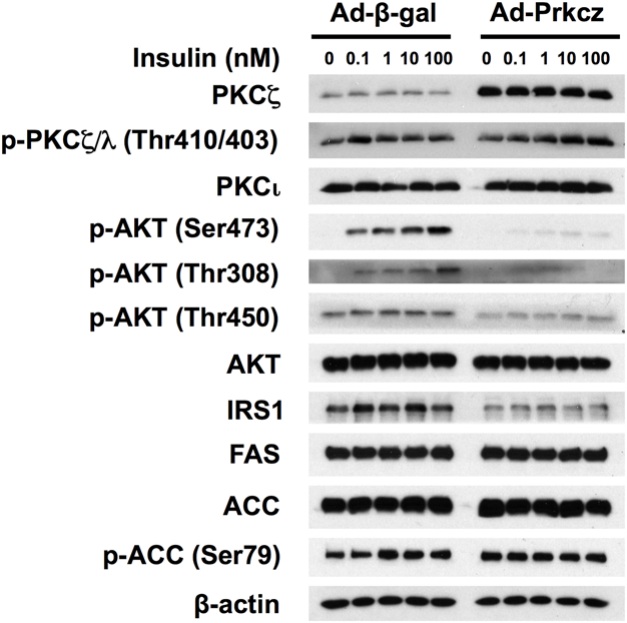
Overexpression of PKCζ attenuated the activation of insulin signaling cascade in primary hepatocytes of ZL rats. Primary hepatocytes of ZL rats fed chow for 8 weeks were isolated and seeded onto dishes as described in the Materials and Methods. Purified Ad-β-gal and Ad-Prkcz were added in ZL primary rat hepatocytes during the overnight pretreatment period to allow the overexpression of β-GAL and PKCζ, respectively. Cells were then incubated in fresh medium A with increasing concentrations of insulin (0 nM to 100 nM) for 15 min. The whole cell lysates were subjected to immunoblot analysis with antibodies against indicated proteins. The figure shows the representative result of three parallel experiments obtained from three sets of animals.

**Fig 5:**
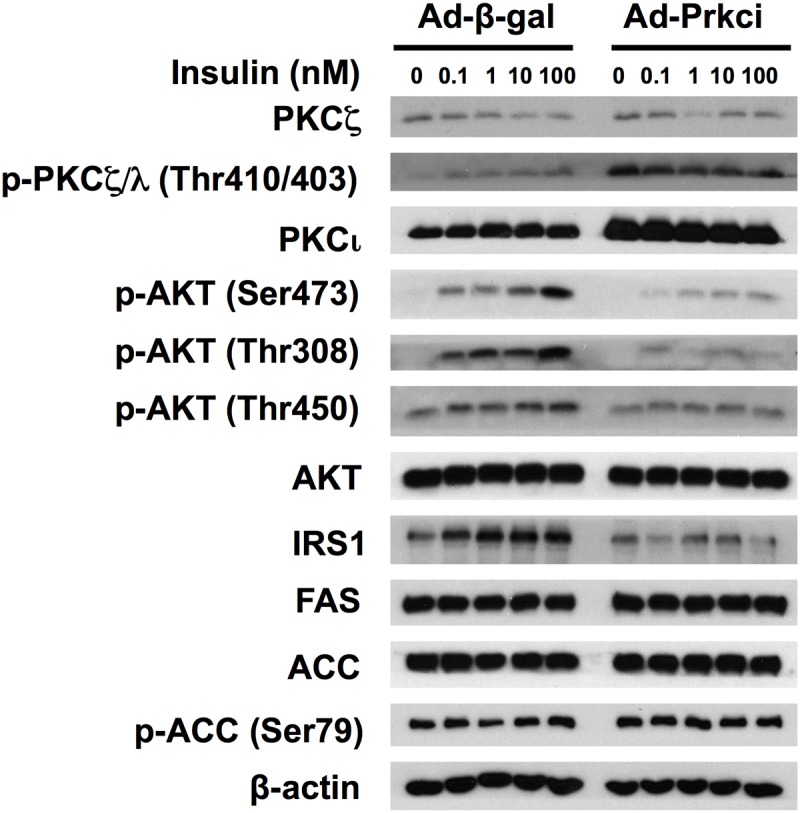
Overexpression of PKCι/λ attenuated the activation of insulin signaling cascade in primary hepatocytes of ZL rats. Primary hepatocytes of ZL rats fed chow for 8 weeks were isolated and seeded onto dishes as described in the Materials and Methods. Purified Ad-β-gal and Ad-Prkci were added in ZL primary rat hepatocytes during the overnight pretreatment period to allow the overexpression of β-GAL and PKCι/λ, respectively. Cells were then incubated in fresh medium A with increasing concentrations of insulin (0 nM to 100 nM) for 15 min. The whole cell lysates were subjected to immunoblot analysis with antibodies against indicated proteins. The figure shows the representative result of three parallel experiments obtained from three sets of animals.

Fig. 4 shows that PKCζ, but not PKCι/λ, protein level was overexpressed by ~9.3 folds in hepatocytes transfected with Ad-Prkcz compared to that with Ad-β-gal. Overexpression of PKCζ did not significantly increase phospho-PKCζ/λ Thr410/403 level. Fig. 5 shows that primary rat hepatocytes transfected with Ad-Prkci manifested ~4.0 fold overexpression of PKCι/λ protein and increased phosphorylation of PKCζ/λ at Thr410/403, compared to that with Ad-β-gal. Unfortunately, we tried and failed to develop a method for the determination of activities of PKCζ and PKCƖ/λ using substrates as reported and available antibodies.

### Overexpression of PKCζ or PKCι/λ attenuated the insulin signaling cascade in ZL primary hepatocytes

Fig. 4 shows that the levels of insulin-induced phosphorylation of AKT at Ser473 and Thr308, and insulin-independent phosphorylation of AKT at Thr450, were diminished in hepatocytes overexpressing PKCζ, but not β-GAL. There was no change of total AKT. The expression levels of IRS1 were also lowered upon the overexpression of PKCζ. Interestingly, based on the densitometry analysis, there was a slight but significant elevation in the levels of PKCι/λ, FAS, ACC or phospho-ACC at Ser79 in the hepatocytes overexpressing PKCζ compared with Ad-β-gal group (S3 Fig.).

Fig. 5 shows that PKCι/λ overexpression did not significantly change the expression levels of PKCζ and ACC in ZL primary hepatocytes. The levels of FAS and phospho-ACC Ser79 were marginally but significantly elevated (S4 Fig.). PKCι/λ overexpression did not change the total AKT level compared with Ad-β-gal group. Similar to that in PKCζ overexpression groups, the levels of IRS1, insulin-induced phospho-AKT at Ser473 and Thr308, and insulin-independent phosphorylation of AKT at Thr450 were significantly decreased in primary rat hepatocytes overexpressing PKCι/λ in comparison to the Ad-β-gal group. These data collectively demonstrate that overexpression of aPKC attenuates the activation of insulin signaling cascade in primary hepatocytes from ZL rats.

### Overexpression of PKCζ or PKCι/ζ impaired the expression levels of insulin-regulated genes in primary hepatocytes from ZL rats

**Fig 6:**
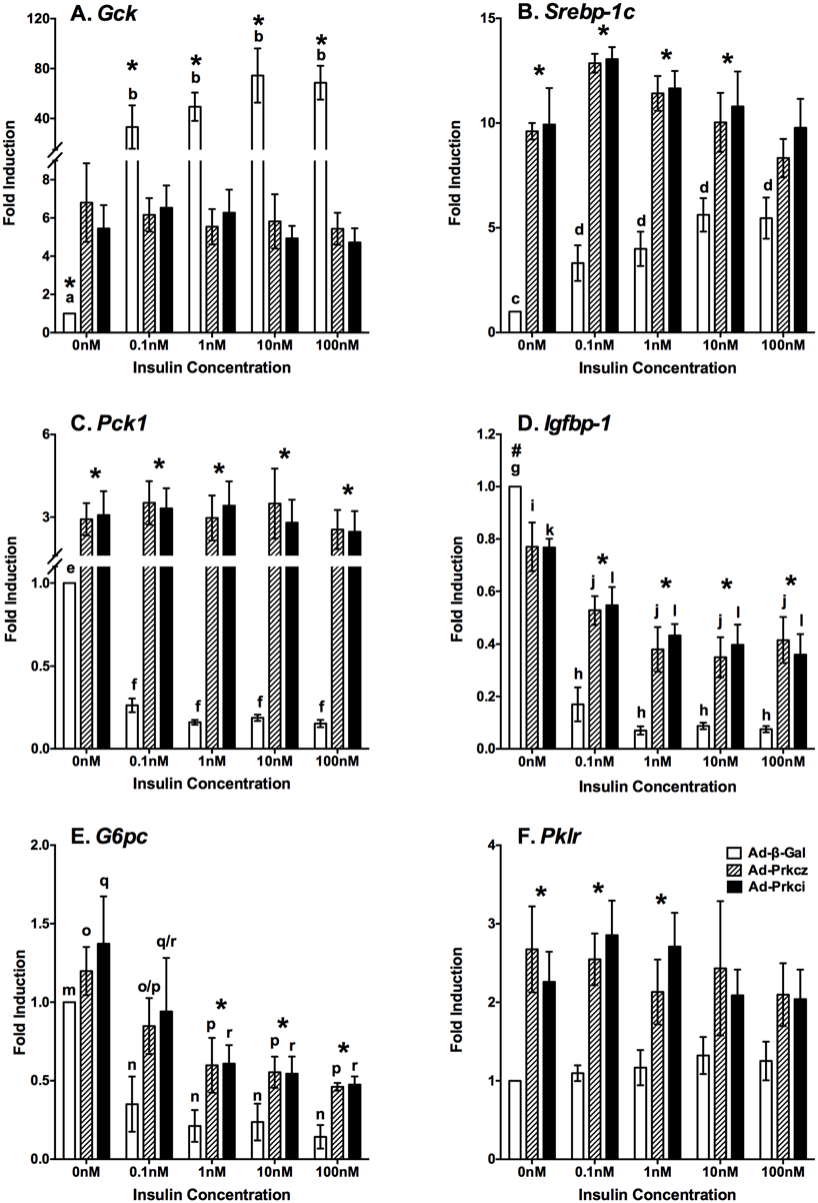
Overexpression of PKCζ and PKCι/λ impaired the insulin-regulated gene expression in ZL primary rat hepatocytes. Primary hepatocytes of ZL rats fed chow for 8 weeks were isolated and seeded onto dishes as described in the Materials and Methods. Purified Ad-β-gal, Ad-Prkcz and Ad-Prkci were added in medium A during the overnight pretreatment period to allow the overexpression of β-GAL, PKCζ and PKCι/λ, respectively. The primary hepatocytes were then incubated in fresh medium A with increasing concentrations of insulin (0nM to 100nM) for 6 hours. Total RNA was extracted, and subjected to real-time PCR analysis. The transcript level of each indicated gene in primary hepatocytes transfected with Ad-β-gal in the absence of insulin treatment was arbitrarily set to 1 for that gene. The mRNA levels of (A) *Gck*, (B) *Srebp-1c*, (C) *Pck1*, (D) *Igfbp-1*, (E) *G6pc* and (F) *Pklr* were expressed as fold inductions (mean ± SEM; n = 4; all p<0.05; a<b, c<d, e>f, g>h, i>j, k>l, m>n, o>p, q>r for comparing the insulin treatment groups of hepatocytes overexpressing Ad-β-gal, Ad-Prkcz or Ad-Prkci using one-way ANOVA; * for comparing fold inductions among Ad-β-gal, Ad-Prkcz and Ad-Prkci groups at the indicated insulin concentrations using one-way ANOVA).

Insulin dose-dependently induced the expression levels of *Gck* (Fig. 6A) and *Srebp-1c* (Fig. 6B) transcripts in Ad-β-gal group. Overexpression of PKCζ or PKCι/λ increased the basal levels of *Gck* and *Srebp-1c* in ZL primary hepatocytes. However, overexpression of PKCζ or PKCι/λ abolished the insulin-induced *Gck* and *Srebp-1c* expressions in ZL hepatocytes.

Insulin dose-dependently suppressed the expression levels of *Pck1* (Fig. 6C), *Igfbp-1* (Fig. 6D) and *G6pc* (Fig. 6E) transcripts in Ad-β-gal group as anticipated. In the absence of insulin, overexpression of PKCζ or PKCι/λ increased or reduced the basal expression levels of *Pck1* (Fig. 6C) or *Igfbp-1* (Fig. 6D) transcript, respectively. However, the insulin-suppressed expression of *Pck1* was abolished, and insulin-suppressed expressions of *Igfbp-1* and *G6pc* were attenuated in hepatocytes overexpressing PKCζ or PKCι/λ, respectively.

Fig. 6F shows that the mRNA levels of *Pklr* in hepatocytes overexpressing -GAL, PKCζ and PKCι/λ were not affected by insulin. However, the expressions levels of *Pklr* in 0-1nM insulin groups were significantly higher in hepatocytes overexpressing PKCζ or PKCι/λ than that overexpressing β-GAL.

## Discussion

Here we show that the expression levels of PKCζ protein and *Prkcz* mRNA are higher in the liver of ZF rats than ZL rats. This elevation remains intact in primary hepatocytes that have been cultured for more than 20 h. Since the insulin-regulated gene expression is impaired in primary hepatocytes from ZF rats, we hypothesize that the alteration of aPKC expressions may play a role in this impairment. Therefore, PKCζ and PKCι/λ are successfully overexpressed using recombinant adenoviruses in ZL primary hepatocytes. The adenoviral-mediated overexpression of PKCζ or PKCι/λ in primary hepatocytes from ZL rats leads to the impairment of insulin-regulated *Gck*, *Srebp-1c*, *Pck1*, *G6pc* and *Igfbp1* expressions (Fig. 6). Overexpression of either PKCζ or PKCι/λ is sufficient to alter basal and insulin-regulated gene expression in hepatocytes from ZL rats.

The impairment of the insulin-regulated gene expression might be attributed to the reduction of of AKT phosphorylations in cells with overexpression of PKCζ or PKCι/λ., Maximal activation of AKT needs phosphorylations of AKT at both Ser473 and Thr308 as indicated in [4]. Therefore, detection of its phosphorylation using specific antibodies have been a indicator of AKT activation status. It was interesting to note that we could not detect phosphorylation of AKT at Thr308 in the liver tissue lysates of both ZL and ZF rats. Here are the possible reasons. First, the in vivo activation of AKT may be moderate, not maximized, which allows further regulation in response to stimuli. Second, there might be relative less AKT protein amount from hepatocytes in the liver tissue preparations, which requires a larger amount of protein per sample for the detection. Third, the phosphorylation of AKT at Thr308 in the liver may be rapidly de-phoshorylated during the time when the animals were euthanized for the collection of tissue samples. Nevertheless, we demonstrated here that in ZL hepatocytes of Ad-β-gal group, insulin dose-dependently induces the levels of phospho-AKT (Ser473 and Thr308) and IRS1 protein. On the other hand, PKCζ or PKCι/λ overexpression attenuates the insulin-induced phosphorylation of AKT, and decreases the protein level of IRS1, which suggests a diminished signaling cascade initiated from IRS1 upon insulin treatment (Figs. 4 and 5). Without insulin treatment, the expression level of total AKT and p-AKT seem to be not different between the Ad-β-gal control and aPKCs overexpression groups (Figs. 4 and 5). However, higher mRNA levels of *Gck*, *Pck1*, *Srebp-1c* and *Pklr* were observed in the aPKCs overexpression groups compared to those in the Ad-β-gal control (Fig. 6). This indicates that other mechanisms may contribute to the elevation of the basal expression of those genes in hepatocytes overexpressing PKCζ or PKCι/λ.

GCK catalyzes the phosphorylation of glucose into glucose-6-phosphate, which is the rate-limiting step of glycolysis in the liver [24]. The induction of the hepatic *Gck* expression depends on the activation of insulin signaling pathway in the liver [11]. PKCζ (Fig. 4) or PKCι/λ (Fig. 5) overexpression diminished phosphorylation of AKT at Ser473 along with the decrease of IRS1 protein expression. It has been reported that aPKCs probably act as negative feedback regulators of insulin signaling pathway by promoting serine phosphorylation and tyrosine de-phosphorylation of IRS proteins both *in vitro* and in cell lines [25–27]. Thus, the reduction of the activation of insulin signaling pathway due to PKCζ or PKCι/λ overexpression may impair the insulin-regulated *Gck* expression. Additionally, the insulin-induced *Gck* expression has been suggested to be mediated by transcription factors, such as hepatocyte nuclear factor 4, forkhead box protein O1, SREBP-1c, liver x receptor α, and peroxisome proliferator-activated receptor γ [28–31]. Since the knowledge about the non-kinase activities and endogenous substrates of PKCζ or PKCι/λ is limited, it is possible that PKCζ or PKCι/λ directly regulate *Gck* expression through controlling the activation of certain transcription factors at its promoter, which increases the *Gck* basal mRNA level.

PEPCK catalyzes the rate limiting enzymatic reaction in gluconeogenesis, by which oxaloacetate is converted into phosphoenolpyruvate [32]. In the liver, the insulin-suppressed *Pck1* expression has been thought to be purportedly mediated through three pathways: (1) the phosphorylation and inactivation of forkhead box proteins by insulin [33,34]; (2) antagonizing the glucagon-induced PPARγ co-activator 1 expression [35]; (3) the phosphorylation of CREB-binding protein via aPKCs [36,37]. Interestingly, insulin-induced activation of aPKCs was shown to suppress *Pck1* expression via this phosphorylation of SREBP-binding protein [36]. However, our results show the induction of *Pck1* expression upon overexpression of PKCζ or PKCƖ/λ in the absence of insulin. The activities of aPKC isoforms are elevated in the liver of individuals with type 2 diabetes [17]. Treatment of hepatocytes from these individuals with aPKC inhibitors decreases the basal transcript levels of gluconeogenic genes [17]. Hepatic inhibitors of PKCƖ have been show to correct the metabolic abnormalities in rodents [18]. Here, aPKCs overexpression not only increases the basal transcript of *Pck1* mRNA, but also abrogates the insulin-suppressed *Pck1* expression. Additionally, aPKCs overexpression attenuates the insulin-suppressed *G6pc* expression. Therefore, our data suggest that aPKCs are prominent positive regulators of hepatic gluconeogenesis.

SREBP-1c is a master regulator of the hepatic expression of lipogenic genes [38]. The insulin-induced *Srebp-1c* expression requires the activation of PI3K, and the mTORC2-mediated phosphorylation of AKT at Ser473 [39]. Based on the use of pharmacological inhibitors, the activation of mTORC1 downstream of AKT is identified as an indispensible step in the induction of *Srebp-1c* [39]. On the other hand, the activation of PI3K subsequently activates aPKC in the liver, which promotes *Srebp-1c* expression and lipogenesis [12,13]. Despite the diminished activation of AKT and the abolished insulin-induced *Srebp-1c* expression, PKCζ or PKCι/λ overexpression increases the basal transcript level of *Srebp-1c* in the primary hepatocytes from ZL rats (Fig. 6C). Interestingly, PKCζ or PKCι/λ overexpression only marginally induced the protein levels of FAS and ACC (Figs. 4 and 5). One possibility is that the elevation levels of transcripts might not have been translated in the presence of overexpression of PKCζ or PKCι/λ. Alternatively, diminished insulin signaling pathway results in decreased activation of p70 S6-kinase, which is required for the SREBP-1c processing to generate nuclear SREBP-1c [40]. Moreover, there might be a lag time between the elevation of *Srebp-1c* mRNA and the increase of SREBP-1c protein, and the down-stream targets. Nevertheless, the protein levels of ACC and are maintained at a high level in the hepatocytes and liver samples of ZF rats (Figs. 1 and 2), demonstrating the metabolic changes associated with these insulin-resistant animals. Further investigations are needed to delineate the sequential events associated with the elevation of the hepatic lipogenesis upon insulin stimulation and activation of AKT and aPKC isoforms.

It is interesting to note that the *Prkcz* mRNA and PKCζ protein, but not that of *Prkci* mRNA and PKCι/λ protein levels are elevated in the liver of ZF rats. Insulin at 1nM and up significantly induces the expression levels of *Prkci* mRNA, a process that is blunted in the presence of PKCζ overexpression. On the other hand, insulin does not affect the expression of *Prkcz* mRNA. Additionally, PKCι/λ overexpression alone does not affect *Prkcz* mRNA expression. However, PKCι/λ overexpression introduced the insulin-mediated suppression of *Prkcz* mRNA expression. These data indicate that there are crosstalks between PKCζ and PKCι/λ pathways, which may mutually regulate the expression of each other at mRNA levels in response to hormonal or nutritional stimuli. The alteration of this mutual regulation mechanism probably is associated with the development of the impairment of insulin signaling cascade. Further studies are warranted.

It is worth noting that, in ZL primary hepatocytes, the overexpression of aPKCs and the attenuated insulin signaling cascade lead to abolishment of insulin-regulated expression of *Gck*, *Pck1* and *Srebp-1c* (Fig. 6). In contrast, ZF rats showed unimpaired phosphorylation of AKT at Ser473 and Thr450 despite increased PKCζ levels (Figs. 1 and 2). This difference may explain that the insulin-regulated expression of *Gck* and *Pck1* is impaired rather than abolished in ZF primary hepatocytes [41]. Additionally, in high-fat diet fed mice, increased basal AKT phosphorylation was observed in the liver with indication for the development of insulin resistance [42]. Additionally, elevated aPKC activity was shown to attenuate AKT-dependent FOXO1 phosphorylation in the liver of diet-induced obesity mice, despite concurrent elevated hepatic AKT activity [43]. These data collectively suggest that activation statuses of both AKT and aPKC are critical in the regulation of hepatic insulin sensitivity.

In summary, we have shown that the expression level of PKCζ is elevated in insulin-resistant animals. Overexpression of PKCζ or PKCƖ/λ in ZL primary hepatocytes reduces the activation of AKT probably through the reduction of IRS1 expression, demonstrating the attenuation of the activation of insulin signal pathway. This alteration of the expression levels of aPKC leads to the impairment of the insulin-regulated gene expression in primary hepatocytes. Our data demonstrate the critical roles of aPKCs in the regulation of hepatic insulin-sensitivity at gene expression level, and provide insights into the development of the insulin resistance and type 2 diabetes.

## Supporting Information

S1 Fig Densitometry analysis of target proteins in the livers of ZL and ZF rats. Densitometry analysis was performed using ImageJ software (NIH). The data for each protein were normalized to β-actin levels in each sample. One-way ANOVA with LSD post-hoc test was used to compare the means. Error bars represent S.E.M. * indicates a p value less than 0.05.
(DOCX)

S2 Fig Ct number of the ribosomal gene 36B4 in ZL hepatocytes transfected with Ad-β-gal, Ad-Prkcz and Ad-Prkci, respectively. Primary hepatocytes of ZL rats fed chow for 8 weeks were isolated and seeded onto dishes as described in the Materials and Methods. Purified Ad-β-gal, Ad-Prkcz and Ad-Prkci were added in medium A during the overnight pretreatment period to allow the overexpression of β-GAL, PKCζ and PKCι/λ, respectively. The primary hepatocytes were then incubated in fresh medium A with increasing concentrations of insulin (0 nM to 100 nM) for 6 hours before total RNA extraction. Total RNA was extracted, and then subjected to real-time PCR analysis with primer pairs for 36B4. The data were the raw Ct number of 36B4 for 0 nM insulin treatment groups (mean ± SEM; n = 4).
(DOCX)

S3 Fig Densitometry analysis of target proteins in ZL hepatocytes transfected with Ad-β-gal or Ad-Prkcz. Densitometry analysis was performed using ImageJ software (NIH). The data for each protein were normalized to β-actin levels in each sample. Two-way ANOVA was used to determine the contribution of insulin treatment and adenovirus overexpression to total variance. Error bars represent S.E.M. * indicates that adenovirus overexpression accounts for a significant percentage of total variance with a p value less than 0.05. a<b, c<d<e, which indicate insulin treatments account for a significant percentage of total variance with a p value less than 0.05.
(DOCX)

S4 Fig Densitometry analysis of target proteins in ZL hepatocytes transfected with Ad-β-gal or Ad-Prkci. Densitometry analysis was performed using ImageJ software (NIH). The data for each protein were normalized to β-actin levels in each sample. Two-way ANOVA was used to determine the contribution of insulin treatment and adenovirus overexpression to total variance. Error bars represent S.E.M. * indicates that adenovirus overexpression accounts for a significant percentage of total variance with a p value less than 0.05. a<b, a’<b’, c<d, e<f, which indicate insulin treatments account for a significant percentage of total variance with a p value less than 0.05.
(DOCX)
