# Supplementary figures and images for: Higher Protein Kinase C ζ in Fatty Rat Liver and Its Effect on Insulin Actions in Primary Hepatocytes
Source: PLoS One. 2015 Mar 30;10(3):e0121890. doi: 10.1371/journal.pone.0121890 (PMC4379029; doi:10.1371/journal.pone.0121890)

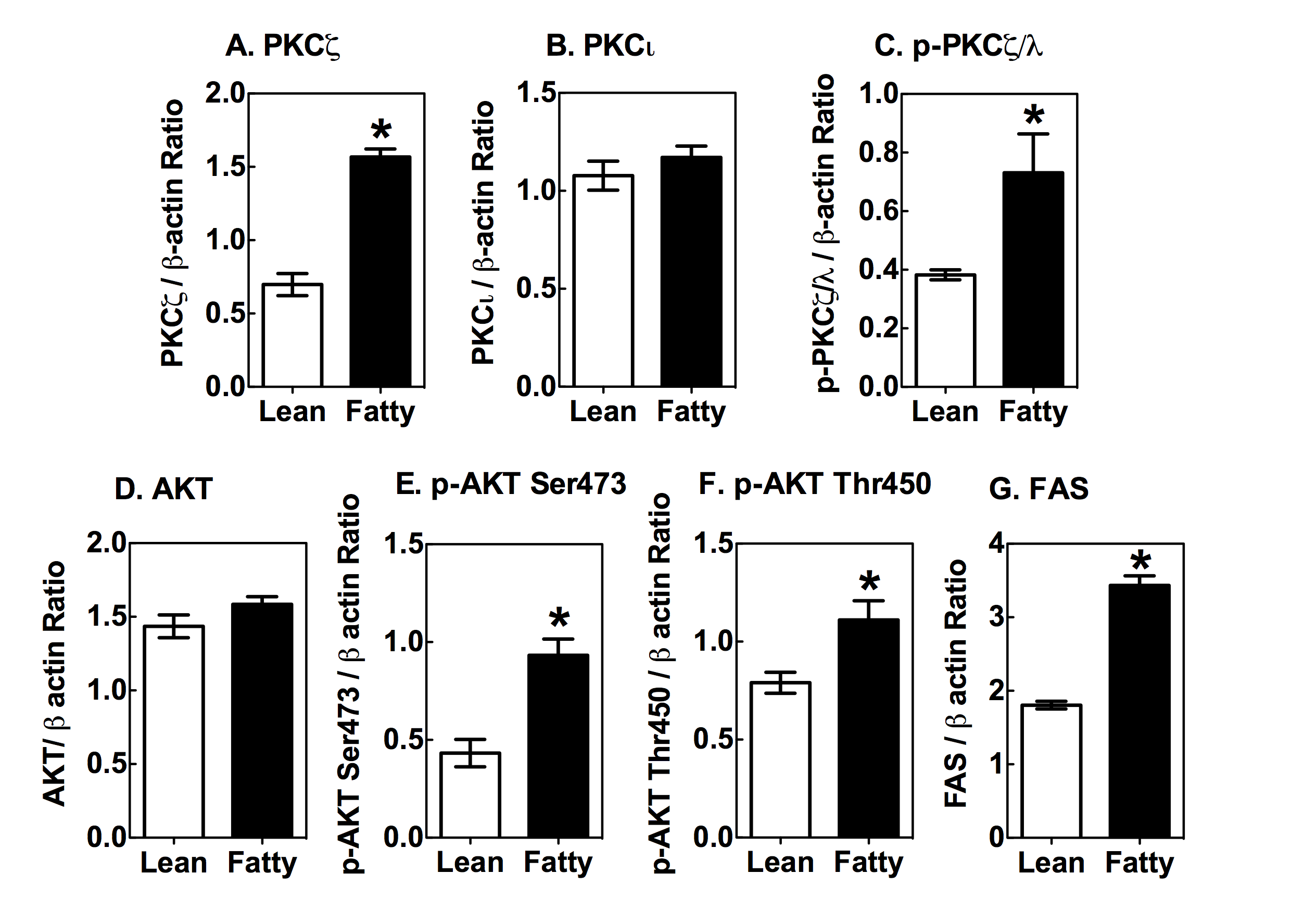

Supplement: S1 Fig — Densitometry analysis was performed using ImageJ software (NIH). The data for each protein were normalized to β-actin levels in each sample. One-way ANOVA with LSD post-hoc test was used to compare the means. Error bars represent S.E.M. * indicates a p value less than 0.05. (DOCX) [file pone.0121890.s001.docx]

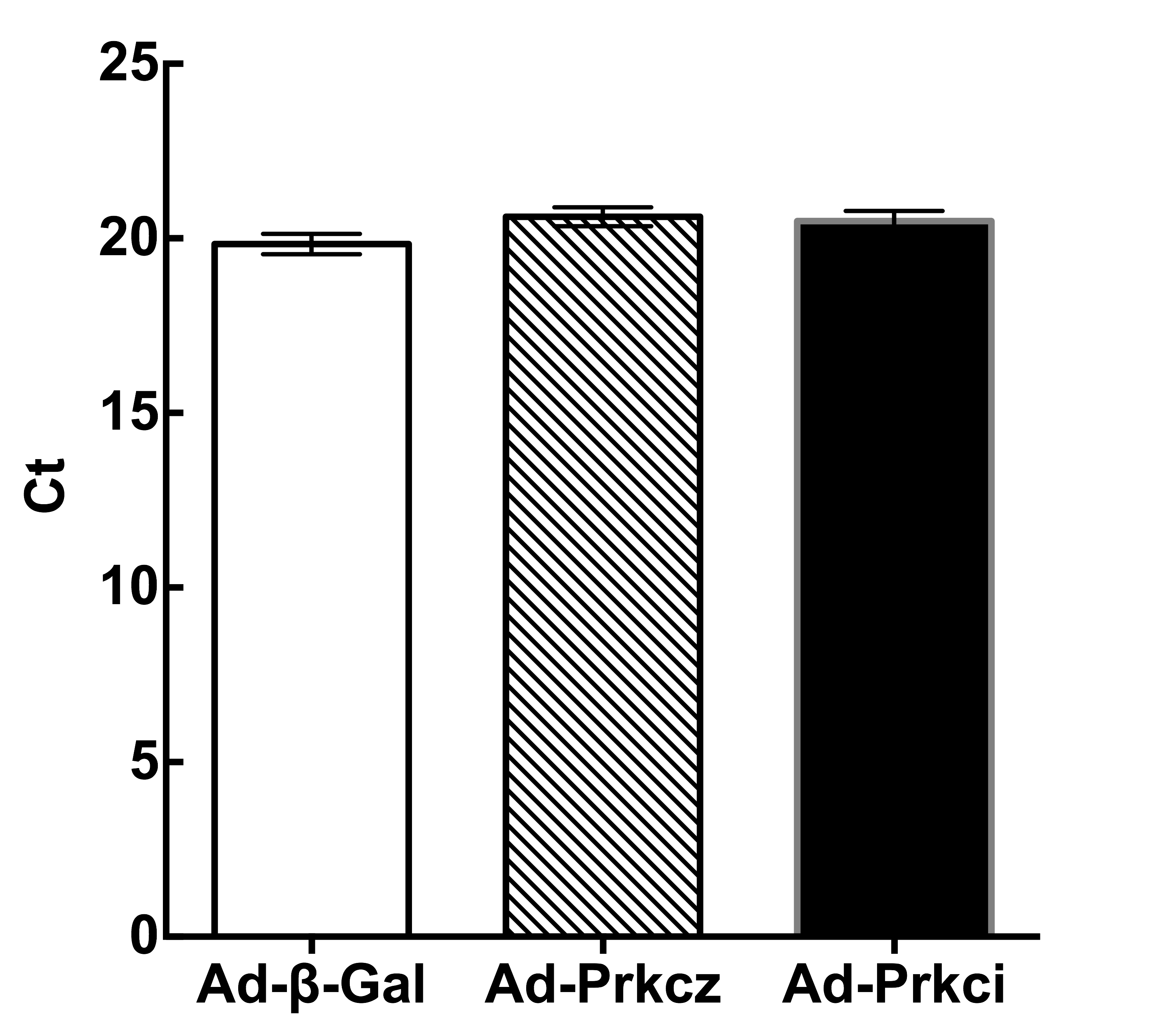

Supplement: S2 Fig — Primary hepatocytes of ZL rats fed chow for 8 weeks were isolated and seeded onto dishes as described in the Materials and Methods. Purified Ad-β-gal, Ad-Prkcz and Ad-Prkci were added in medium A during the overnight pretreatment period to allow the overexpression of β-GAL, PKCζ and PKCι/λ, respectively. The primary hepatocytes were then incubated in fresh medium A with increasing concentrations of insulin (0 nM to 100 nM) for 6 hours before total RNA extraction. Total RNA was extracted, and then subjected to real-time PCR analysis with primer pairs for 36B4. The data were the raw Ct number of 36B4 for 0 nM insulin treatment groups (mean ± SEM; n = 4). (DOCX) [file pone.0121890.s002.docx]

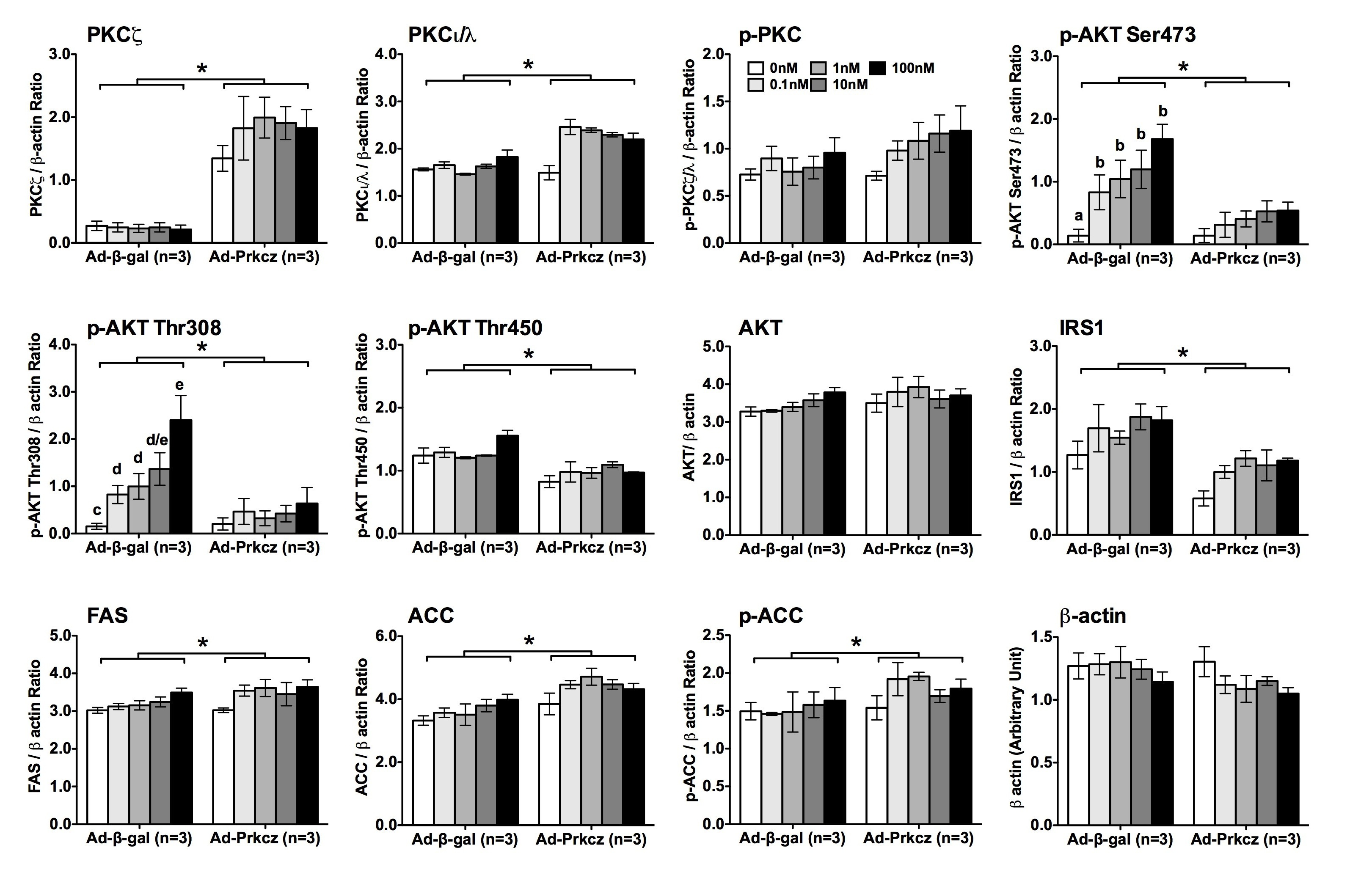

Supplement: S3 Fig — Densitometry analysis was performed using ImageJ software (NIH). The data for each protein were normalized to β-actin levels in each sample. Two-way ANOVA was used to determine the contribution of insulin treatment and adenovirus overexpression to total variance. Error bars represent S.E.M. * indicates that adenovirus overexpression accounts for a significant percentage of total variance with a p value less than 0.05. a<b, c<d<e, which indicate insulin treatments account for a significant percentage of total variance with a p value less than 0.05. (DOCX) [file pone.0121890.s003.docx]

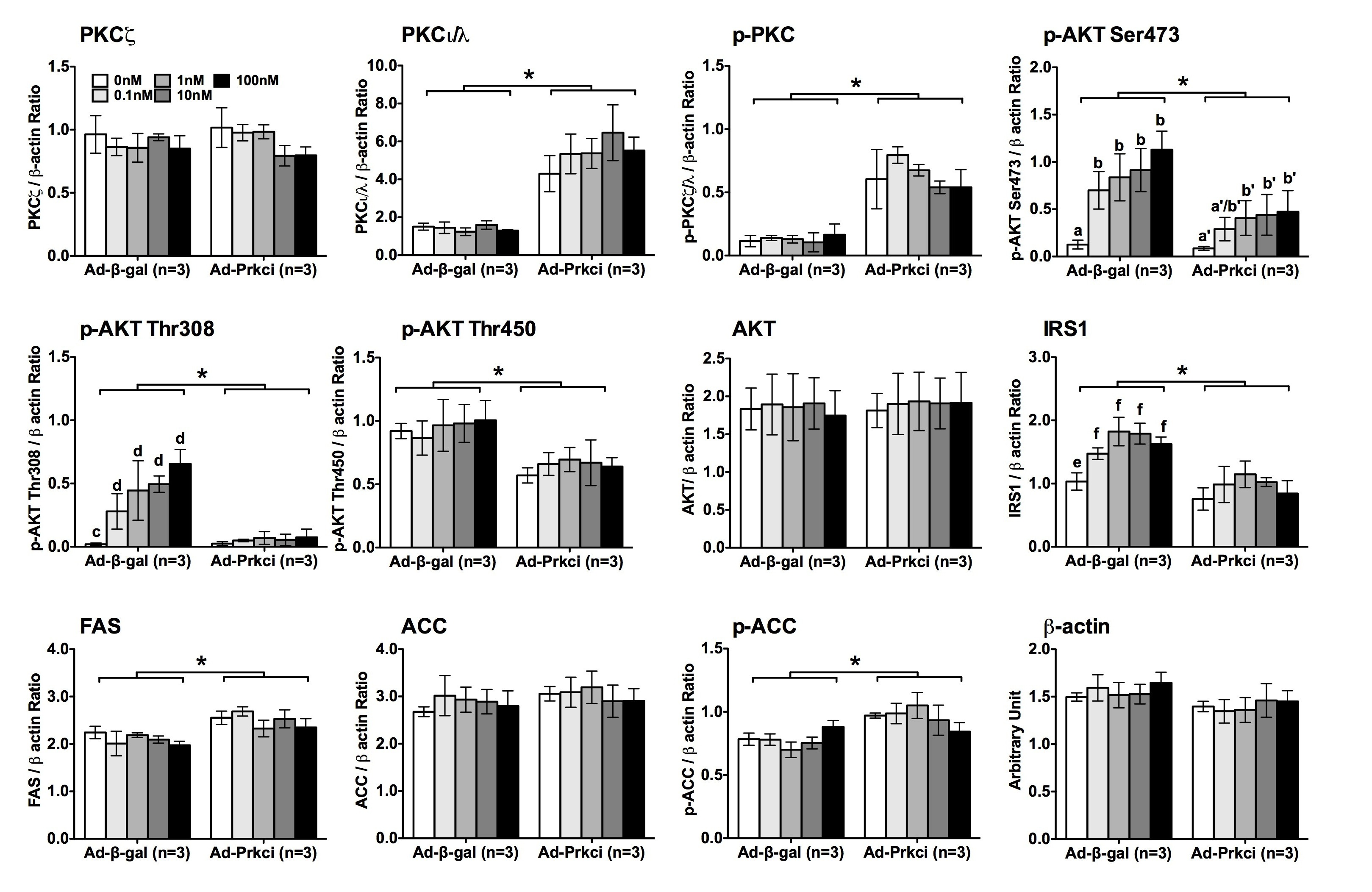

Supplement: S4 Fig — Densitometry analysis was performed using ImageJ software (NIH). The data for each protein were normalized to β-actin levels in each sample. Two-way ANOVA was used to determine the contribution of insulin treatment and adenovirus overexpression to total variance. Error bars represent S.E.M. * indicates that adenovirus overexpression accounts for a significant percentage of total variance with a p value less than 0.05. a<b, a’<b’, c<d, e<f, which indicate insulin treatments account for a significant percentage of total variance with a p value less than 0.05. (DOCX) [file pone.0121890.s004.docx]
